# Effects of lipid composition on photothermal optical coherence tomography signals

**DOI:** 10.1117/1.JBO.25.12.120501

**Published:** 2020-12-23

**Authors:** Mohammadhossein Salimi, Martin Villiger, Nima Tabatabaei

**Affiliations:** a York University, Lassonde School of Engineering, Department of Mechanical Engineering, Toronto, Canada; b Harvard Medical School, Massachusetts General Hospital, Wellman Center for Photomedicine, Boston, Massachusetts, United States

**Keywords:** photothermal optical coherence tomography, photothermal phenomena, optical coherence tomography, lipid, molecular contrast imaging, atherosclerosis

## Abstract

**Significance:** Photothermal optical coherence tomography (PT-OCT) has the promise to offer structural images coregistered with chemical composition information, which can offer a significant impact in early detection of diseases such as atherosclerosis.

**Aim:** We take the first step in understanding the relation between PT-OCT signals and the endogenous tissue composition by considering the interplay between the opto-thermo-physical properties of tissue as a function of its lipid composition and the ensuing effects on the PT-OCT signals.

**Approach:** Multiparameter theoretical estimates for PT-OCT signal as a function of composition in a two-component lipid–water model are derived and discussed. Experimental data from various concentrations of lipid in the form of droplets and injections under bovine cardiac muscle align with theoretical predictions.

**Results:** Theoretical and experimental results suggest that the variations of heat capacity and mass density with tissue composition significantly contribute to the amount of optical path length difference measured by OCT phase.

**Conclusion:** PT-OCT has the potential to offer key insights into the chemical composition of the subsurface lipid pools in tissue; however, the interpretation of results needs to be carried out by keeping the nonlinear interplay between the tissue of opto-thermo-physical properties and PT-OCT signals in mind.

Optical coherence tomography (OCT) is an interferometric optical imaging method which utilizes low-coherence light to form micrometer-resolution images of optical scattering media (e.g., biological tissues) in two and three dimensions.^1^ OCT systems are widely used in medical practice and research, specifically for interrogation of tissues that are incompatible with excisional biopsy (e.g., the eye, arteries, or nervous tissues).^1^^,^^2^ Conventional OCT measures the path length of elastically back-scattered light and provides images of tissue structure rather than chemical composition (scattering versus absorption of light). Consequently, OCT is quite sensitive to structural abnormalities induced at the early stages of diseases. However, in the cases where structural abnormalities are insufficient for clearly diagnosing the disease stage (e.g., atherosclerosis^3^^,^^4^ or early dental caries^5^), conventional OCT imaging often results in false positive readings, limiting the reliability and diagnostic value of OCT.

Photothermal optical coherence tomography (PT-OCT) is a functional extension of OCT with the promise to overcome the nonspecific nature of conventional OCT by forming three-dimensional (3D) images based on both scattering and absorption of light.^6^ In PT-OCT, an intensity-modulated photothermal (PT) laser with a wavelength in the absorption band of a molecule of interest is added to the conventional OCT system. In such a configuration, absorption of the PT laser by the molecule of interest induces a localized modulated temperature field (aka thermal wave field). This thermal wave field, in return, yields modulated thermo-elastic expansion resulting in modulated variation in the local refractive index. These phenomena ultimately lead to the modulation of the optical path length (OPL) at the modulation frequency of the PT laser with a modulation amplitude on the order of tens of nanometers. Since the OCT phase has enough sensitivity to measure such small variations in OPL, PT-OCT has the potential to offer coregistered structural and molecular information through the OCT signal’s amplitude and phase, respectively. To date, nanoparticles have been employed in PT-OCT as contrast agents to perform molecular-specific detection in human^7^ and rabbit^8^ tissues *ex vivo* as well as to detect cancer cells *in vitro.*^9^ More importantly, the feasibility of label-free imaging with PT-OCT has been demonstrated by measuring blood oxygen saturation in vessel phantoms,^10^ melanin distribution in zebra-fish eye,^11^ and lipids in human coronary arteries.^12^ Although these studies demonstrate the potential of PT-OCT for resolving and possibly quantifying molecules of interest in 3D, they assume a linear relation between the concentration of the molecule of interest and the PT-OCT signal. Although this assumption may be met for exogenous agents or molecules dissolved in aqueous media,^10^^,^^11^ in the more general case, variation in the composition of a tissue not only alters its optical properties but also induces changes in other physical properties (e.g., mass density and thermal properties). In this paper, we take the first step in understanding the relation between tissue composition and PT-OCT signals by considering the interplay between the opto-thermo-physical properties of tissue as a function of its composition. We develop a theoretical model estimating the PT-OCT response in a two-component tissue-like sample. We then present experimental PT-OCT results of mayonnaise (mayo)-ultrasound gel mixtures at various component ratios. Mayo was chosen to mimic the lipid-rich necrotic-core material present in atheromatous coronary atherosclerotic lesions.^13^^,^^14^ Mayo is primarily composed of lipids, which provide an absorption signature that can be targeted with PT-OCT, and its lipid composition is similar to that of atherosclerotic plaques.^13^^,^^14^ Since the composition and lipid content play a critical role in determining the propensity of a plaque to rupture,^15^ PT-OCT may offer the prospect to leverage the same contrast mechanism as that used by near-infrared spectroscopy^16^ and photoacoustic imaging^17^^–^^19^ (i.e., absorption of lipid) for plaque composition imaging, albeit with much finer resolution.

Figure 1(a) shows schematic representation of processes that take place upon absorption of the PT laser in a PT-OCT system (e.g., absorption of 1210-nm laser by lipid–water mixture^17^). Absorption of intensity-modulated PT light results in production of heat $Q_{\mathrm{in}}$, which subsequently diffuses into the molecular matrix, forming a localized thermal wave field modeled by the bio-heat equation. When the PT laser spot size is small compared to the absorption depth ($1/\mu_{a}$), the bio-heat equation is dominated by transverse/radial heat transfer.^20^ In such cases, the amplitude of temperature modulation, ΔT at a given depth and at the center of the PT beam is expressed by^20^.

$$\Delta T=\frac{P\mu_{a}}{4\alpha \pi \rho c}\;\ln(1+\frac{t_{L}\alpha }{\omega^{2}/8}),$$ (1)

$$\overset{\text{power series};\;\deg<2}{\to }\Delta T∼\frac{2Pt_{L}}{\pi \omega^{2}}\times \frac{\mu_{a}}{\rho c}.$$ (2)

Here, P is the PT-laser power, $\mu_{a}$ is the medium’s absorption coefficient at the PT-laser wavelength, α is its thermal diffusivity, ρ is its mass density, c is its specific heat capacity, ω is the waist of the PT laser beam, and $t_{L}$ is the laser exposure time, taken as half of the PT laser modulation cycle, assuming a square modulation. By expanding the logarithm in Eq. (1) as a power series and neglecting terms of degree two and more, ΔT can be estimated as a simpler expression only containing the product of variables, Eq. (2). For a given PT-OCT setting, only parameters c, ρ, and $\mu_{a}$ in Eq. (2) are affected by the sample’s composition. Considering a linear variation of these parameters in a two-component mixture,^21^^,^^22^ the value of a given parameter can be modeled as

$$\zeta =(\zeta_{2}-\zeta_{1})r+\frac{\left(\zeta_{2}+\zeta_{1}\right)}{2}=\zeta_{\text{range}}r+\zeta_{\text{mean}},\;-0.5\leq r\leq 0.5\;\text{and}\;\zeta_{C}=\frac{\zeta_{\text{range}}}{\zeta_{\text{mean}}}.$$ (3)

Here, ζ is the concentration-dependent material parameter (e.g., ρ), r is the relative concentration with respect to a 1:1 mixture, and $\zeta_{1}$, $\zeta_{2}$ are the values of the material parameters of pure individual components, respectively. $\zeta_{C}$ is the contrast of the material property in the two-component mixture and indicates the sensitivity of the parameter to variation of concentration. Using Taylor expansion, ΔT can then be expressed in terms of concentration-dependent material parameters as:

$$\Delta T(r)=\frac{2Pt_{L}}{\pi \omega^{2}}\times \frac{\mu_{\text{mean}}}{\rho_{\text{mean}}c_{\text{mean}}}\times (1+[\mu_{C}-\rho_{C}-c_{C}]r+M(\mu_{C},\rho_{C},c_{C})r^{2}+\ldots ).$$ (4)

**Fig. 1:**
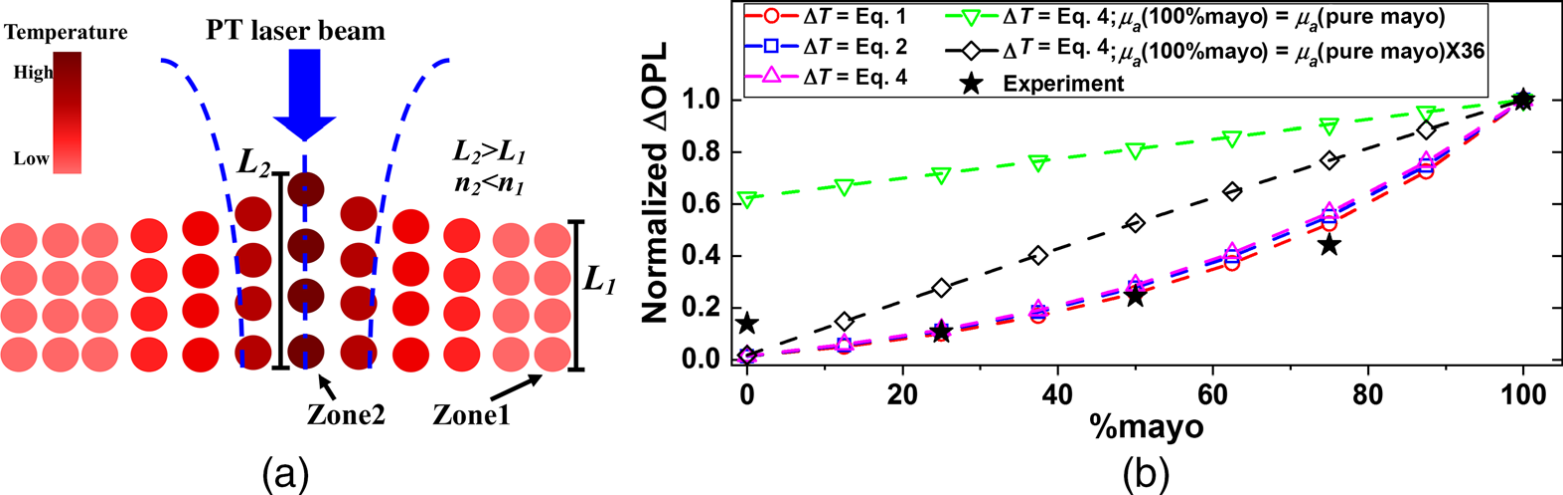
(a) Schematic presentation of sequence of physical processes taking place in PT-OCT upon absorption of PT light. (b) Numerical and experimental results for ΔOPL as a function of concentration of mayonnaise in the sample.

Equation (4) lies at the heart of this work and suggests that the temperature variation resulting from PT-laser illumination depends on nontrivial interactions of multiple physical parameters. Only when PT-OCT imaging is carried out on aqueous samples containing relatively low amounts of exogenous agents or dissolved molecules^10^^,^^11^ will ΔT depend linearly on their concentration. That is so because in these scenarios, changes in concentration do not lead to considerable change in density and specific heat capacity (i.e., $\rho_{C}≅c_{C}≅0$), making the coefficients of nonlinear terms in Eq. (4) negligible. This linear prediction aligns well with the results reported to date on different concentrations of indocyanine green (ICG)-water mixtures^23^ or those on blood oxygen saturation.^10^ Variation of concentration in biological tissue compounds (e.g., lipid), on the other hand, results in considerable changes in density and specific heat capacity, leading to nonlinear variation of ΔT with concentration. The degree of this nonlinearity is determined by the contrasts of the component’s properties $\zeta_{C}$.

Once ΔT is induced, the volume and the local refractive index of the molecular matrix change [Fig. 1(a)] and the optical path length difference (ΔOPL) as a function of relative concentration r can be found as^24^

$$\Delta \mathrm{OPL}(r)=\overset{L_{1}}{\underset{L_{0}}{\int }}n(T,r)dl=n(T_{0}+\Delta T)L_{1}-n(T_{0})L_{0}=[n(r)+\frac{dn}{dT}(r)\Delta T(r)][L_{0}+L_{0}\beta (r)\Delta T(r)]-n(r)L_{0}\to \Delta \mathrm{OPL}(r)=L_{0}\{[n(r)\beta (r)+\frac{dn}{dT}(r)]\Delta T(r)+[\frac{dn}{dT}(r)\beta (r)]\Delta T{\left(r\right)}^{2}\},$$ (5)

→ΔOPL∝ΔT. (6)

Here, $T_{0}$ is the initial temperature of the sample, dn/dT is its thermo-optic coefficient, and β is its linear thermal expansion coefficient. Considering the range of values of parameters of Eq. (5) in biological systems (see Table 1), the quadratic term of ΔT is negligible compared to the linear term. Therefore, the ΔOPL measured through the phase signal of OCT is directly proportional to variations in temperature ΔT.

**Table 1:** Opto-thermo-physical properties of components used in simulations.

Property (unit)	Water	Mayo
Absorption coefficient $\mu_{a}$ ($m^{-1}$)	100^17^	160^17^
Density ρ ($\mathrm{kg}/m^{3}$)	1000	910
Specific heat c (J/kg K)	4184	2450^25^^,^^26^
Thermal effusivity e (${\mathrm{Ws}}^{1/2}/m^{2}\;K$)	1588	740^21^
Refractive index n	1.34^24^	1.49^27^
Thermo-optic coefficient $\frac{dn}{dT}$	$-91\times 10^{-6}$^24^	$-531\times 10^{-6}$^27^
Linear thermal expansion coefficient β ($K^{-1}$)	$100\times 10^{-6}$^24^	$1000\times 10^{-6}$^28^

To get a better understanding of the predictions of the developed model, we simulated the PT-OCT responses of various ratios of mayo–water mixtures. The opto-thermo-physical properties used for pure mayo and water (i.e., $\zeta_{1}$ and $\zeta_{2}$) are depicted in Table 1. The simulation results of the normalized ΔOPL as a function of mayo concentration are plotted in Fig. 1(b). In this plot, simulations are normalized with respect to the corresponding PT-OCT signals at 100% mayo concentration in order to make the results independent of the variation of PT laser parameters with depth (e.g., intensity or beam waist). When considering a mixture in which the properties of the solvent are dominant ($\rho_{C}≅c_{C}≅0$), the change in the concentration predominantly induces a change in the absorption coefficient of the PT light. Under this condition, the nonlinear terms of Eq. (4) become negligible, resulting in a linear relation between ΔT [and consequently ΔOPL; Eq. (6)] and mayo concentration, as experimentally observed in solvent-dominant samples by other groups.^10^^,^^23^ However, when the sample is considered as a compound of mayo and water, the material properties are determined by the weight percentage of its components, Eq. (3). Since there is a pronounced difference between the opto-thermo-physical properties of mayo and water (Table 1), parameters $\rho_{c}$, $c_{c}$, and $\mu_{c}$ in Eq. (4) are all considerable, leading to the nonlinear variation of ΔT with mayo concentration. Furthermore, the refractive index, its temperature dependence, and the expansion coefficient in Eq. (5) are also concentration-dependent (see Table 1) and contribute to the nonlinearity of ΔOPL seen in Fig. 1(b). Consequently, unlike the linear behaviors reported to date on samples with exogenous agents or dissolved molecules in aqueous media,^10^^,^^11^ the general variation of ΔOPL with the concentration of one tissue component is nonlinear. Another notable point in Fig. 1(b) is the small ΔOPL predicted for water (i.e., %mayo = 0) despite water’s moderate absorption at 1210 nm.^17^ The reason behind the small ΔOPL for water is the large heat capacity of water acting against the rise of the temperature as well as the cancellation of contributions of thermal expansion, n(r)β(r), and the thermo-optic coefficient, $\frac{dn}{dT}(r)$, to OPL changes. The small ΔOPL of water is specifically important for PT-OCT of biological tissues that generally have high water content because it helps with the detection of PT-OCT signatures of waterless tissue components above the small PT-OCT signal baseline from water. Therefore, in PT-OCT, the large absorption coefficient of a component does not necessarily lead to a strong PT-OCT signal and, in fact, the optimization of PT-OCT experiments needs to take place by considering the interplay between the opto-thermo-physical properties of tissue.

To experimentally verify our theoretical predictions, we used a spectral-domain PT-OCT system (Fig. S1 in the Supplementary Material), employing broadband light of a superluminescent diode centered at 1310 nm (±75  nm at 10 dB; Exalos, Switzerland), a 2048-pixel line scan camera spectrometer with a maximum acquisition rate of ∼147  kHz (Wasatch Photonics; USA), and an intensity modulated single-mode PT laser (1210 nm; power on sample 18 mW). The axial and lateral resolutions of OCT in tissue were measured as 8.5 and 10  μm, respectively. Using a common-path reference, a displacement error of 3 nm at a signal-to-noise ratio of 35 dB was measured, which is close to that of a shot-noise-limited system.^29^ However, PT-OCT experiments were carried out without the phase reference using the absolute OCT phase (i.e., subject to inherent phase drifts of interferometer, laser scanning system, and other instabilities^29^). The OCT and PT beams are concentric and coscanned with an FWHM spot size of 8  μm. PT-OCT experiments were carried out at an A-line rate of 21.6 kHz. Each M-scan consisted of 5000 data points acquired in ∼230  ms. The PT signal was sinusoidal and modulated at 500 Hz, corresponding to the fundamental frequency of the square signal assumed for the theoretical derivation. The signal processing steps on a graphics processing unit (GPU) for forming PT-OCT images included: applying fast Fourier transformation (FFT) on the acquired spectra to get OCT amplitude and phase images, forming M-mode phase signals of each depth location, applying FFT on the M-mode phase signals for calculating PT-OCT image pixel amplitude using Eq. (7),^30^ removing outlier pixels, and subtracting base line

$$\mathrm{amp}(Z)=\frac{|p(z,f_{0})|\lambda_{0}}{4\pi^{2}f_{0}\Delta t}.$$ (7)

Here, |p| is the normalized FFT amplitude of the phase signal at the PT laser modulation frequency of $f_{0}$, $\lambda_{0}$ is the center wavelength of the OCT laser, and Δt is the acquisition time for one A-line.

To prepare various dilutions of the lipid–water compound, droplets of mayo and ultrasound gel with appropriate weight ratios were dispensed in a Petri dish and stirred well to make homogenous samples at five different weight concentrations of mayo (100%, 75%, 50%, 25%, and 0%). The scattering of the 0% mayo sample was adjusted to those of other mayo concentrations using titanium dioxide powder. For the dilution experiments, the average of the pixels in the PT-OCT B-mode images were normalized to that of the 100% mayo sample, as seen in Fig. 1(b). In addition, to examine the efficacy of the system in detecting lipid in tissue, lipid-rich atherosclerotic plaque was mimicked by injecting 95% mayo compound into fresh normal bovine cardiac muscle. A similar sample was also prepared using 40% mayo compound to examine the PT-OCT system’s sensitivity to lipid concentration in tissues. The thickness of the cap above the lipid pool for the 40% and 95% mayo simulated plaques varied between 110 and 360  μm and 140 to 280  μm, respectively. These simulated cap dimensions are comparable to those encountered in vulnerable coronary plaques.^31^

Figure 2 depicts results obtained from samples at various mayo concentrations. Conventional OCT images of (a)–(e) cannot discern between different concentrations of mayo/lipid. The root cause of this limitation is the fact that conventional OCT forms images based on elastic scattering of light, which originates from discontinuities in the materials’ refractive index rather than based on the presence of a characteristic molecular marker of the disease. Hence, tissues with very different compositions may have very similar OCT structural signatures. A case in point is the identification of thin-cap fibroatheromas, characterized by the presence of a necrotic core and extracellular lipid, and their differentiation from less vulnerable pathological intimal thickenings.^3^ Both plaque types bear similar structural features when imaged with intravascular OCT, complicating the identification of plaques at increased risk of causing future adverse events. The PT-OCT images of (f)–(j), on the other hand, clearly show the strong direct correlation of PT-OCT signal strength with concentration of mayo/lipid. The PT-OCT images of Fig. 2 qualitatively suggest a nonlinear variation of PT-OCT signal with concentration. The normalized average PT-OCT signals of each mayo concentration is plotted in Fig. 1(b) together with the simulated results. The nonlinear variation of PT-OCT signal with %mayo is evident in this plot, as predicted by the theoretical model. Another key observation is the significant underestimation of ΔOPL contrast between 0% and 100% mayo when only considering the absorption coefficient ($\mu_{a}$) to be concentration-dependent in the model. In such a single-variable model, a 36-times higher absorption coefficient is needed for mayo to achieve the ΔOPL contrast seen in experimentation. The reason behind this discrepancy is that, in practice, both $\rho_{C}$ and $c_{C}$ play an intensifying role based on Eq. (4) in producing a larger ΔT compared to the case where concentration dependence of $\rho_{C}$ and $c_{C}$ are neglected. Also note that while the non-linear trend of variation of normalized ΔOPL with %mayo is reasonably close to the predicted theoretical values in the 25% to 100% range, the experimental PT-OCT signal deviates from the predicted theoretical trend at 0% (i.e., water) because the measured PT-OCT signal is dominated, in this case, by the noise floor in the OCT phase channel. The noise floor is likely limited by phase drift of the interferometer and could be reduced with a common-path reference. Using a fixed reflection signal in the sample arm just above the sample as a phase reference could also significantly reduce the phase noise, while being amenable to downstream translation into catheter-based systems (e.g., via phase reference taken from surface of the catheter ball lens).

**Fig. 2:**
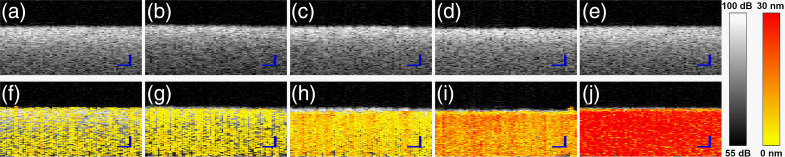
OCT images of samples with mayonnaise concentration: (a) 0%, (b) 25%, (c) 50%, (d) 75%, and (e)100%; PT-OCT images of samples with mayonnaise concentration (f) 0%, (g) 25%, (h) 50%, (i) 75%, and (j) 100%. Scale bars=50  μm.

Imaging results of the artificial lipid-rich plaques are shown in Fig. 3. In conventional OCT images, the boundary of the 40% mayo sample can clearly be identified in the OCT image of (a) while the boundary of the 95% mayo, (b), is diffuse, similar to those seen in OCT imaging of the lipid pool in human atherosclerotic plaques.^32^ In either case, information about the composition of the tissue cannot be obtained from the conventional OCT images. The PT-OCT images of (d) are from the sample with 95% mayo injection (i.e., similar lipid content as atherosclerotic plaques^13^^,^^14^). The lipid-rich pool is clearly identified in this image. The PT-OCT image of (c) is from the 40% mayo injection. Here, the subsurface diluted lipid pool cannot be reliably recognized as the PT-OCT signals are within the range of noise floor. While the lipid content here is 42% of that of the sample in (d), the resulting PT-OCT signal is only about 20% of that of the sample in (d). This nonlinear variation of PT-OCT signal with concentration is in complete alignment with the theoretical predictions of our model. Results of Fig. 3 indicate that PT-OCT has potential for offering key insights into the chemical composition of the subsurface lipid pools in tissue; however, the interpretation of results needs to be carried out with care and by keeping the nonlinear interplay between the opto-thermo-physical properties and PT-OCT signals in mind.

**Fig. 3:**
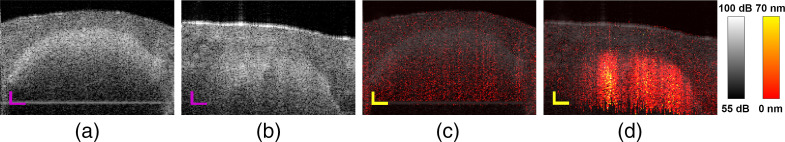
OCT image of the plaque phantom with mayonnaise concentration: (a) 40% and (b) 95%; PT-OCT image of the plaque phantom with mayonnaise concentration (c) 40% and (d) 95% (scale bar=100  μm).

In summary, this work provides a theoretical model and a preliminary experimental study demonstrating the feasibility of obtaining insights into the chemical composition of biological tissues using PT-OCT. The developed model and experimental results suggest that the relation between PT-OCT signals and the concentration of an absorbing component in the tissue is nonlinear, and that the degree of this nonlinearity is determined by the contrasts of a component’s opto-thermo-physical properties $\zeta_{C}$. Another key conclusion of this work is that in PT-OCT, a large absorption coefficient of a component does not necessarily lead to a strong PT-OCT signal. Instead, the strength of the PT-OCT signal is highly dependent on the interplay between the opto-thermo-physical properties of the tissue, all of which are concentration-dependent. Our experimental results suggest the possibility of obtaining insight into the lipid content of tissue-like samples and may offer a pathway toward refined assessment of atherosclerotic plaques in the future.
