# Supplementary material for: Effects of lipid composition on photothermal optical coherence tomography signals
Source: J Biomed Opt. 2020 Dec 23;25(12):120501. doi: 10.1117/1.JBO.25.12.120501 (PMC7757902; doi:10.1117/1.JBO.25.12.120501)
Supplement: Supplementary file 1 [file JBO_025_120501_SD001.pdf]

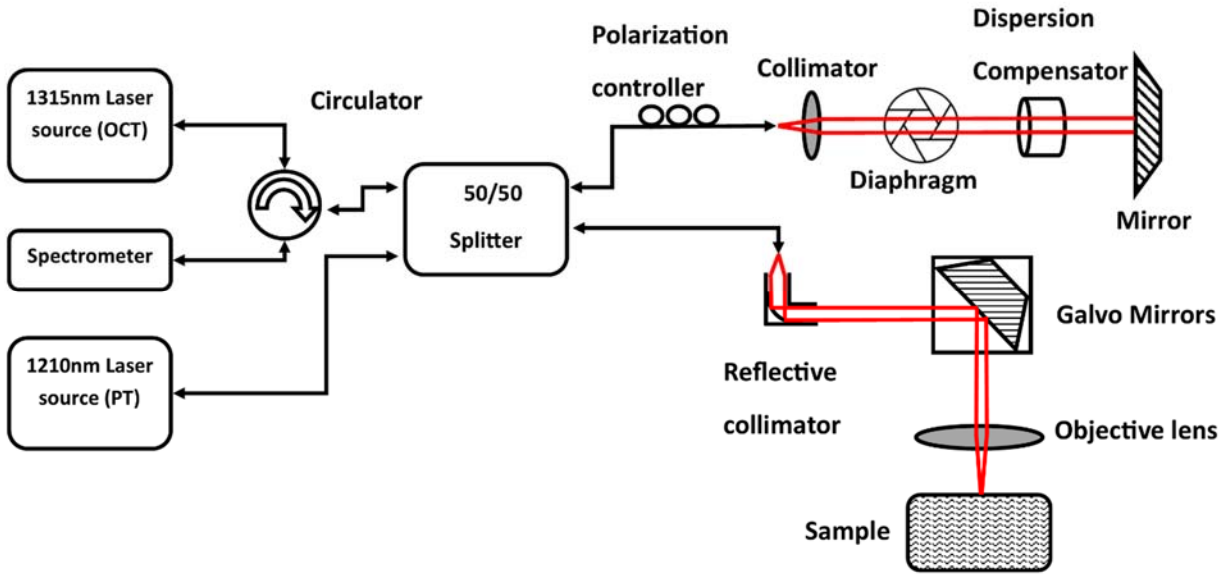

Fig. S1 Schematic of the photothermal optical coherence tomography (PT-OCT) setup used in this study.
